# Loss of DNA methylation at imprinted loci is a frequent event in hepatocellular carcinoma and identifies patients with shortened survival

**DOI:** 10.1186/s13148-015-0145-6

**Published:** 2015-10-15

**Authors:** Sumadi Lukman Anwar, Till Krech, Britta Hasemeier, Elisa Schipper, Nora Schweitzer, Arndt Vogel, Hans Kreipe, Ulrich Lehmann

**Affiliations:** Institute of Pathology, Medizinische Hochschule Hannover, Carl-Neuberg-Str. 1, 30625 Hannover, Germany; Department of Gastroenterology, Medizinische Hochschule Hannover, Carl-Neuberg-Str. 1, 30625 Hannover, Germany; Present address: Department of Surgery, Faculty of Medicine Universitas Gadjah Mada, Yogyakarta, 55281 Indonesia

**Keywords:** Hepatocellular carcinoma, Imprinting, DNA methylation, Pyrosequencing

## Abstract

**Background:**

Aberrant DNA methylation at imprinted loci is an important molecular mechanism contributing to several developmental and pathological disorders including cancer. However, knowledge about imprinting defects due to DNA methylation changes is relatively limited in hepatocellular carcinoma (HCC), the third leading cause of cancer death worldwide. Therefore, comprehensive quantitative DNA methylation analysis at imprinted loci showing ~50 % methylation in healthy liver tissues was performed in primary HCC specimens and the peritumoural liver tissues.

**Results:**

We found frequent and extensive DNA methylation aberrations at many imprinted loci in HCC. Unsupervised cluster analysis of DNA methylation patterns at imprinted loci revealed subgroups of HCCs with moderate and severe loss of methylation. Hypomethylation at imprinted loci correlated significantly with poor overall survival (log-rank test, *p* = 0.02). Demethylation at imprinted loci was accompanied by loss of methylation at *LINE-1*, a commonly used marker for global DNA methylation levels (*p* < 0.001). In addition, we found that loss of methylation at imprinted loci correlated with the presence of a *CTNNB1* mutation (Fisher’s exact test *p* = 0.03). Re-analysis of publically available genome-wide methylation data sets confirmed our findings. The analysis of benign liver tumours (hepatocellular adenoma (HCA) and focal nodular hyperplasia (FNH)), the corresponding adjacent liver tissues, and healthy liver tissues showed that aberrant DNA methylation at imprinted loci is specific for HCC.

**Conclusions:**

Our analyses demonstrate frequent and widespread DNA methylation aberrations at imprinted loci in human HCC and identified a hypomethylated subgroup of patients with shorter overall survival.

**Electronic supplementary material:**

The online version of this article (doi:10.1186/s13148-015-0145-6) contains supplementary material, which is available to authorized users.

## Background

Genomic imprinting represents a deviation from Mendelian laws in which gene expression is regulated to originate only from one allele, either the paternal or the maternal one [1]. Establishment of imprinting takes place during gametogenesis and plays an important role in the regulation of embryogenesis and foetal development [2]. The allele-specific expression is regulated by epigenetic mechanisms especially DNA methylation. In the genomic context, imprinted genes are commonly located in clusters [3]. Each imprinted gene or cluster is surrounded by differentially methylated regions (DMR) wherein methylation appears also in a parent-specific pattern [4]. Regulation of imprinted genes or clusters is controlled by these DMRs in which the patterns are transmitted from male and female gametes to the zygote, reprogrammed during peri-implantation and tissue differentiation, and then firmly maintained throughout somatic development and in adult tissues [3]. Aberrant regulation during imprinting establishment has been associated with several pathological disorders including cancer. Moreover, embryogenesis and carcinogenesis often share common features especially in the involved genes, signalling pathways, as well as the regulatory mechanisms [5].

Many imprinted genes are suggested to play a crucial role in driving the oncogenic switch or suppressing the tumour development. Therefore, deregulation of their expression has also been implicated in various human cancers [6, 7]. Imprinting defects at 11p15.5 locus in Beckwith-Wiedemann syndrome (BWS) confer the patients with high risk of cancer compared to the general population [8]. The first description of DNA methylation-mediated loss of imprinting in cancer is also described in this *H19-IGF2* locus in Wilms’ tumour [9]. Although the mechanisms for imprint defects at the *H19-IGF2* locus vary among different tumour types, gain of methylation at the maternal allele is the most common feature. Hypermethylation at *H19*-DMR causes biallelic expression of *IGF2* accompanied by silencing of *H19* [10]. Loss of imprinting in *P73* can be caused by genetic deletion as reported for neuroblastoma [11] or by aberrant DNA methylation as reported for leukaemia and lymphoma [12]. Genetic deletion of *ARHI* is reported in breast and ovarian cancer [13]. Since then, several other imprinted genes are implicated in carcinogenesis of various malignancies.

In hepatocellular carcinoma (HCC), the fifth most frequent cancer worldwide and the third leading cause of cancer related death, relatively little is known about the dysregulation of DNA methylation at imprinted loci. Most of the studies in HCC related to imprinting defects focused only on a single locus, namely *IGF2*/*H19*. Our initial efforts to study epigenetic instability of imprinted genes in HCC have revealed frequent DNA methylation aberrations resulting in biallelic expression and allele switching at the *DLK1-MEG3* imprinting cluster [14] and identified the well-known tumour suppressor gene *RB1* as a new target for imprint dysregulation in human HCC [15].

Currently, 97 human genes are confirmed to be imprinted [16] leaving the possibility that more imprinted genes contribute to the development of liver malignancy a likely scenario. Analysis of various imprinted loci to determine basal DNA methylation levels in several healthy tissues including the liver has been performed [17]. We therefore extended our analysis to imprinted loci demonstrating approximately 50 % (35–65 %) methylation in healthy liver samples as shown by Woodfine et al. [17]. Using our HCC cohort, frequent and extensive DNA methylation aberrations at imprinted loci could be observed and used for HCC classification. A subgroup of HCC showing widespread loss of methylation at imprinted loci correlates significantly with poor survival identifying imprint instability as a potentially new prognostic marker in HCC.

During the course of this study, Lambert et al. [18] published a survey of DNA methylation changes at imprinted loci in HCC. This study, which is based on the re-analysis of previous data from the same group [19], nicely complements our data and is discussed in detail below.

## Results

### Aberrant DNA methylation in imprinted loci in HCC specimens

**Fig. 1:**
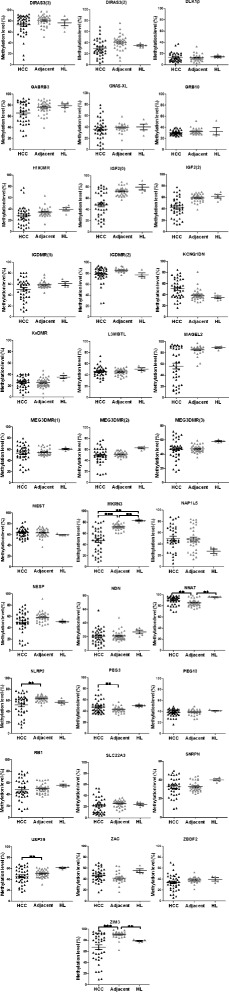
Aberrant DNA methylation at multiple imprinted loci in primary HCC. All individual methylation values quantified with high-resolution pyrosequencing from 40 primary HCC specimens, 34 adjacent liver specimens, and 5 healthy liver tissues are displayed with *scatter plots. Black triangles* represent HCCs and *grey triangles* the adjacent liver tissues. The results of the Mann Whitney *U* test are indicated: **p < 0.01; ***p < 0.001. Additional file 1: Table S1 contains a complete compilation of the results of all statistical calculations

A total of 34 differentially methylated regions (DMRs) described to regulate genomic imprinting in humans and displaying allele-specific DNA methylation in healthy human liver [17] were analysed in primary HCC specimens (*n* = 40), the corresponding adjacent liver tissues (*n* = 34), and healthy liver samples (*n* = 5) using high-resolution quantitative pyrosequencing. The very low variability in the five unrelated control specimens represents the “healthy ground state” of DNA methylation at the loci under study in normal hepatocytes and demonstrates the tight control of the maintenance of DNA methylation patterns at imprinted loci under physiological conditions. Compared to the peritumoural liver tissues and healthy liver specimens, differential DNA methylation in HCC was shown in altogether 25 out of 34 DMRs (Fig. 1 and Table 1). At 14 loci, a substantial gain of methylation was found, whereas a strong loss of methylation was detected at 23 loci, with 11 loci showing gains as well as losses of DNA methylation in different patient samples. This complex pattern is only discernible if the results for each sample are analysed and displayed individually as in Fig. 1. A few loci display already in the adjacent liver specimens clearly discernible alterations in DNA methylation (*DIRAS3(2)*, *NAP1L5*, *MAGEL2*, and *GRBRB3*) supporting the concept that loss of proper regulation at imprinted loci is an early event in the development of human cancer [20]. Since the ground state of DNA methylation at imprinted loci is 50 %, tumour tissue specimens may display gains or losses of methylation. This might lead to the situation that the mean DNA methylation level in tumour specimens is not statistically significantly different from the mean value in healthy tissue despite the presence of substantial deregulation of DNA methylation. This scenario is exemplified by the *RB1* locus (see Fig. 1, second part, fourth row). Therefore, we tested (employing the F-test) also whether the variances in DNA methylation levels differ between the three groups (tumour, adjacent, healthy liver). For the majority of loci, the variance of DNA methylation levels in tumour specimens is significantly different (*p* < 0.0001, see Additional file 1: Table S1 for details) from the variance in the adjacent non-tumourous tissue indicating widespread and pronounced epigenetic instability at imprinted loci in human HCC.

**Table 1:** Gains and losses of DNA methylation at imprinted loci in HCC

		Tumor			Adjacent		
		Loss	Gain	Loss + gain	Loss	Gain	Loss + gain
DIRAS3(3)	1p31	+					
DIRAS3(2)	1p31	++	++	•	++	++	•
ZBDF2	2q33	++	+				
NAP1L5	4q22	++	++	•	++	++	•
ZAC	6q24	(+)			(+)		
SLC22A3	6q26	+	+	•			
GRB10	7p12						
PEG10	7q21						
MEST	7q32						
H19DMR	11p15.5	++	+	•			
IFG2(0)	11p15.5	++					
IGF2(2)	11p15.5	++					
KCNQ1DN	11p15		++				
KvDMR	11p15	(+)			(+)		
RB1	13q14	++	++	•			
DLK1p	14p32						
IGDMR(1)	14q32	++					
IGDMR(2)	14q32	+					
MEG3DMR(1)	14q32	++	+	•			
MEG3DMR(2)	14q32	++	+	•			
MEG3DMR(3)	14q32	++	+	•			
MKRN3	15q11	++					
MAGEL2	15q11	++			+		
NDN	15q11	(+)					
SNRPN	15q11	++	+	•			
GABRB3	15q11	++			+		
NLRP2	19q13	++	(+)	(•)			
PEG3	19q13		+				
USP29	19q13	+ +					
ZIM3	19q13	+ +					
NNAT	20q11						
L3MBTL	20q13		(+)				
GNAS-XL	20q13	++	++	•			
NESP	20q13	++	++	•			
	Sum	23 (+3)	14 (+2)	11	4 (+2)	2	2

The DNA methylation levels for all loci presented in Fig. 1 are classified after visual inspection of the data. The loci are arranged according to their chromosomal localisation

(+) slight alterations**,** + alterations, ++ strong alterations,• gain + loss at the same locus

From Table 1, it is discernible that most alterations in DNA methylation affect whole chromosomal regions involving several imprinted genes and DMRs simultaneously, e.g., on chromosome 11p15, 14q32, or 15q11. However, at closer inspection, a complex picture becomes apparent. At chromosome 11p15, for example, the *H19* DMR displays gains and losses, the *IGF2* DMRs only losses, whereas *KCNQ1DN* displays only gains of DNA methylation.

### DNA methylation at imprinted loci and global DNA methylation

**Fig. 2:**
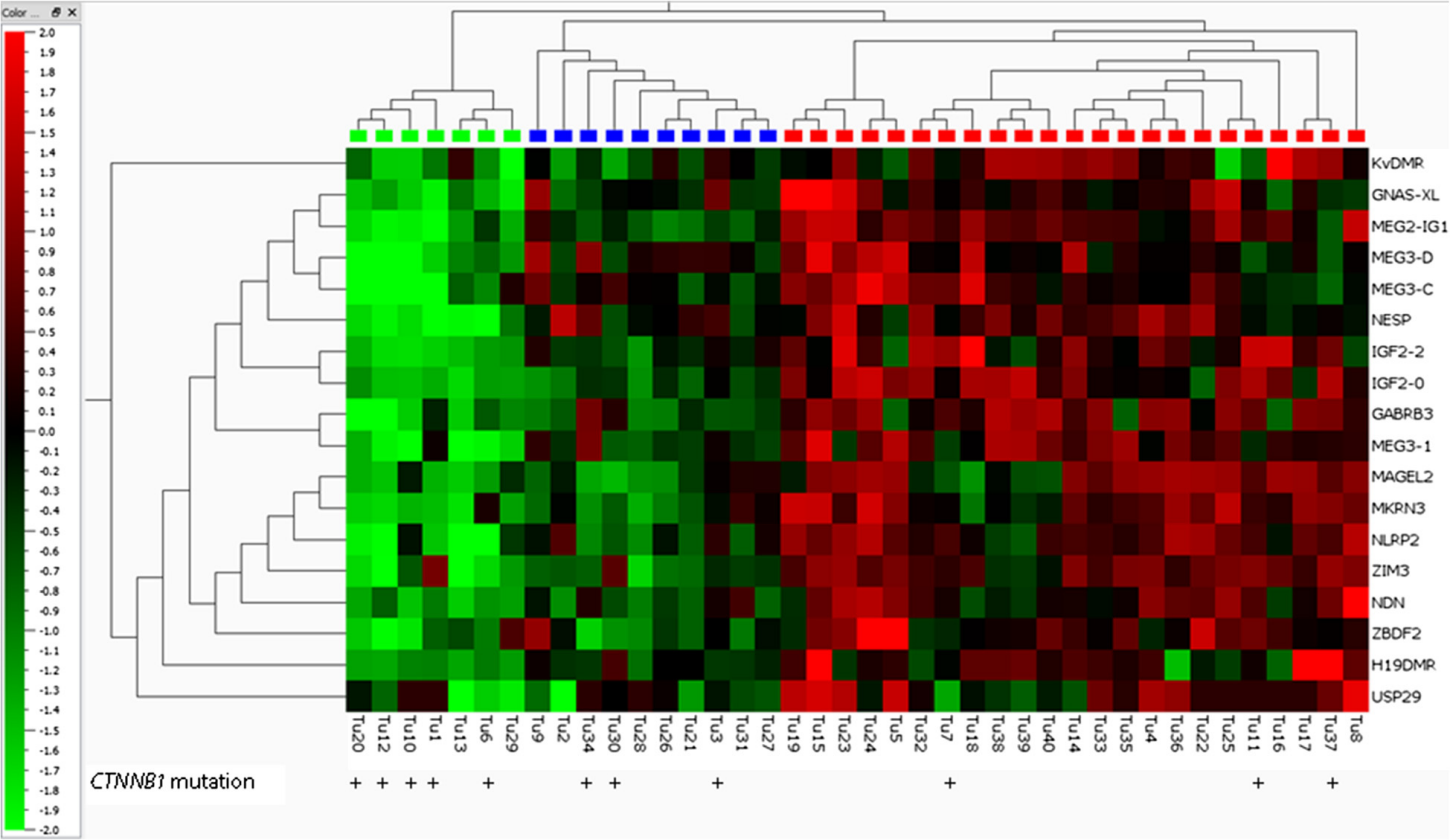
Cluster analysis of methylation levels at imprinted loci. DNA methylation patterns at imprinted loci separate HCC into three subgroups. Unsupervised clustering of methylation levels at imprinted loci was performed. *Upper panel*: *red* = gain of methylation, *blue* = moderate loss of methylation, and *green* = severe loss of methylation. *Lower panel:* distribution of *CTNNB1 (β-catenin)* mutations

**Fig. 3:**
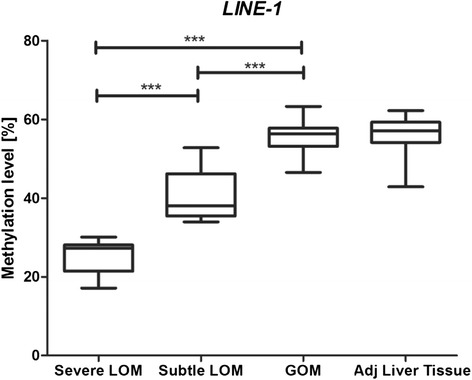
Aberrations of DNA methylation at imprinted loci and global DNA methylation levels. Loss of methylation at imprinted loci is accompanied by reduced DNA methylation at *LINE-1* sequences which serves as a surrogate marker for global DNA methylation level [21]. DNA methylation at *LINE-1* sequences is significantly lower in the subgroup with subtle loss of methylation. The difference is even more pronounced in the subgroup displaying severe loss of methylation at imprinted loci. ***p < 0.001

Unsupervised cluster analysis of quantitative DNA methylation levels in our HCC cohort revealed three distinct subgroups: one displaying marked hypomethylation, a second displaying only subtle hypomethylation, and a third characterized by hypermethylation (Fig. 2). In the next step, global DNA methylation was analysed in every patient sample employing the measurement of *LINE-1* methylation levels as surrogate marker for global DNA methylation levels [21, 22]. The group with only subtle losses of DNA methylation at imprinted loci already demonstrated a significant decrease in *LINE-1* methylation compared to the hypermethylated group (Fig. 3). The HCC group characterized by gains of DNA methylation at imprinted loci showed no change in global DNA methylation level if compared with adjacent liver tissue specimens and healthy liver samples (Additional file 2: Figure S1).

These observations argue against a simple direct connection between global DNA methylation and methylation patterns at imprinted loci and support a model in which these mechanisms are regulated independently from each other.

### Re-analysis of genome-wide DNA methylation analysis at imprinted loci in primary HCC specimens

In order to validate the above described findings, we re-analysed publicly available data of genome-wide DNA methylation analyses in HCC. Two methylom data sets, i.e., Neumann et al. [23] and Shen et al. [24], were retrieved and re-analysed for DNA methylation changes at imprinted loci. Methylation profiles of imprinted genes from both cohorts demonstrated patterns very similar to our findings (Additional file 3: Figure S2). Performing cluster analysis of methylation profiles of imprinted loci contained within these genome-wide DNA methylation data sets, also three subgroups could be identified: heavy and subtle loss of methylation as well as hypermethylation. As a surrogate marker for global DNA methylation levels, the mean *β* values from all CpG sites contained within the Illumina 27K methylation array (excluding the sex chromosomes) were calculated and compared among the three subgroups. Significantly reduced global DNA methylation levels (measured as mean *β* values) were already observed in the group displaying only subtle hypomethylation at imprinted loci (Additional file 4: Figure S3). There was no significant difference between the global DNA methylation level in the group characterized by gain of DNA methylation at imprinted loci on the one hand and healthy liver samples and adjacent liver tissues on the other hand, nicely confirming the complex relationship between global DNA methylation and methylation patterns at imprinted loci observed in our cohort.

### *CTNNB1* mutations and the correlation with methylation at imprinted loci

Mutations of the *CTNNB1* gene have been previously described in a subgroup of HCCs with extensive gain of DNA methylation at tumour suppressor loci [25]. In our cohort, we found that 33 % (11/40) of HCCs harboured activating beta-catenin mutations. However, different from the previous study by Nishida et al. [25], in our cohort, *CTNNB1* mutations were enriched in HCC samples showing loss of DNA methylation at imprinted loci (Fig. 2, Fisher’s exact test *p* = 0.03).

### DNA methylation patterns of imprinted loci in benign liver tumours

To clarify whether aberrant DNA methylation at imprinted loci is cancer cell specific, methylation analysis was performed in benign hepatocellular adenoma (HCA) that show minimal tendency for malignant transformation [26] as well as in focal nodular hyperplasia (FNH), characterized by a strong but benign increase in cell proliferation [27]. None of the benign liver tumour samples and also none of the corresponding adjacent liver tissues showed aberrant DNA methylation at any of the imprinted loci under study (Additional file 5: Figure S4). This demonstrates that DNA methylation aberrations at imprinted loci are specific events in the process of malignant transformation of hepatocytes and not due to a mere increase in cell proliferation which might reduce the fidelity of the maintenance of DNA methylation patterns.

### Methylation of imprinted loci and overall survival

**Fig. 4:**
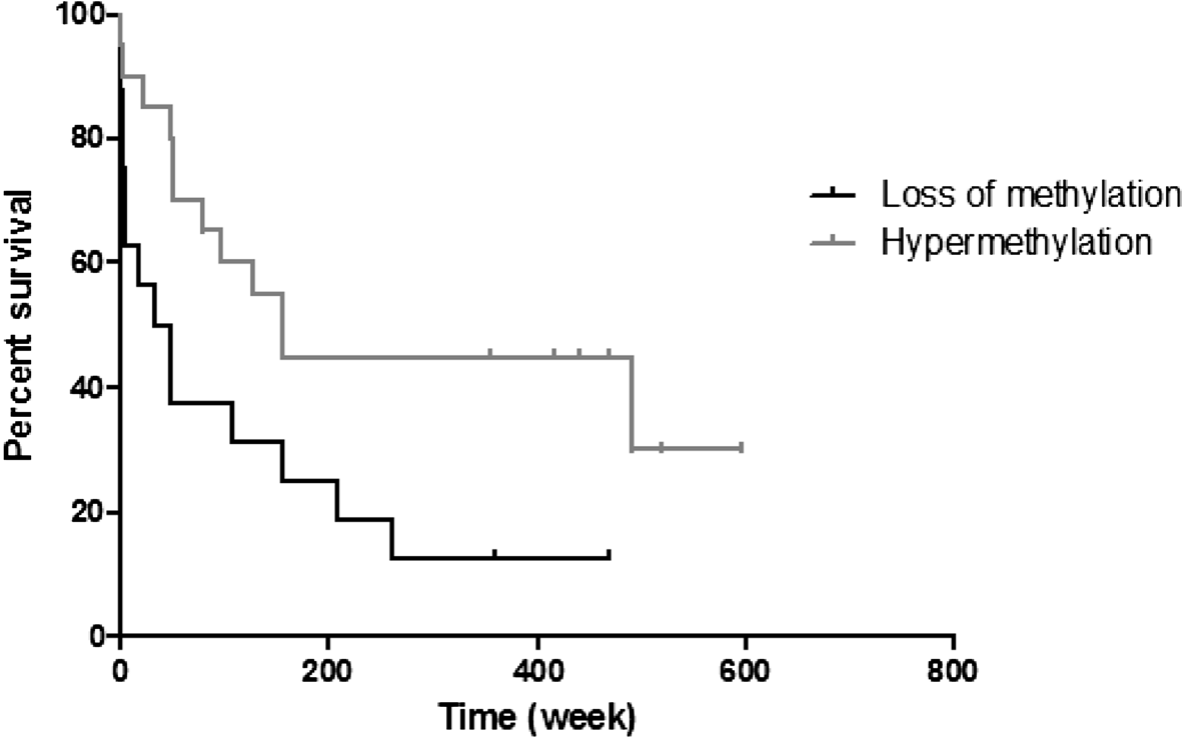
Survival analysis. The overall survival of HCC patients displaying hypomethylation at imprinted loci is significantly lower than in patients with retention of imprint methylation. The Kaplan-Meier plot shows survival of two groups (loss and gain of methylation at imprinted loci), log-rank test *p* = 0.02, median survival 41 and 156 weeks, respectively

Given the ability of methylation profiles at imprinted loci to divide HCCs reproducibly in independent data sets into three distinct classes, we then ascertained the clinical relevance of this finding. Comparing HCC cases showing loss of methylation with the hypermethylated subgroup revealed that those with loss of methylation showed a significantly shortened survival (Fig. 4, log-rank test, *p* = 0.02). Patient age was not significantly different between hypo- and hypermethylated subgroups arguing against different age-distribution as a confounding factor.

## Discussion

Epigenetic instability has been demonstrated as a hallmark of cancer. During the development and progression of cancers, epigenetic aberrations are shown to occur in much higher frequency than genetic alterations [28]. DNA methylation and genomic imprinting are reprogrammed during foetal development, and misregulation of these processes has been associated with several pathological processes including cancer [29]. In HCC, as one of the major types of human cancer, only relatively few studies have addressed DNA methylation aberrations at imprinted loci so far (see [14] and references therein). Studies of imprinting in HCC are mostly dealing with a single locus, i.e., *IGF2-H19*. Here, by analysing multiple imprinted loci, we showed extensive DNA methylation aberrations in primary HCC specimens.

Using DNA methylation patterns at imprinted loci, we demonstrated sub-classification of HCC in which loss of methylation correlates with poor prognosis. This provides the first evidence that DNA methylation changes at imprinted loci can be potentially useful to predict clinical outcomes in HCC and complements recently described molecular classification systems [30]. These findings also provide crucial evidence that in addition to genomic profiling [31], epigenomic profiling is necessary for a comprehensive understanding of the molecular alterations underlying the development of human HCC.

At four loci (*DIRAS3(2)*, *NAP1L5*, *MAGEL2*, and *GRBRB3*), distinct changes in DNA methylation are already detectable in the peritumoural adjacent liver tissue. Already more than 60 years ago, it has been proposed that genetic changes take place in precancerous cells generating a pool of cells from which the malignantly transformed cancer cells arise, a phenomenon termed “field cancerization” [32]. For several malignancies, also epigenetic field defects preceding the development of full blown malignancy have been described [33, 34]. So far, these studies did not examine systematically the involvement of imprinted loci in the liver [35]. Therefore, future studies have to address the expression of the above mentioned loci in precancerous liver conditions like cirrhosis or chronic hepatitis.

From Table 1, it is obvious that the majority of changes in DNA methylation at imprinted loci are represented by loss of methylation. However, this does not always lead to an increase of mRNA expression as one might expect from the usually repressive effect of DNA methylation. Loss of DNA methylation at imprinted loci can lead to reduction in mRNA expression due to increased expression of anti-sense RNAs or interfering RNAs, as shown for the *RB1* locus [15, 36].

Generalized loss of methylation that predominantly affects repetitive DNA elements has been associated with genetic instability [22]. Demethylation at the repetitive elements can initiate retrotransposition of the transposable elements causing deregulation of the neighbouring genes, attenuation of cell cycle checkpoint control, and abrogation of DNA repair systems [37, 38]. Therefore, aggressive tumour behaviour and poor survival are more evident in the subgroup of gastric cancer with hypomethylation at repetitive elements [39].

Recently, Lambert et al. [18] reported also on DNA methylation aberrations at imprinted loci in human HCC. The results are based on the re-analysis of previously published methylation data from the same group [19] obtained by the GoldenGate™ methylation array from Illumina. Despite a different perspective (Lambert et al.: risk factor exposure, this study: survival), a different conceptual approach (Lambert et al.: hypothesis-free analysis of all loci represented on the Illumina GoldenGate™ array, this study: hypothesis-driven analysis of only DMRs displaying 50 % methylation in human healthy liver tissue) and a different methodological basis (Illumina GoldenGate versus pyrosequencing) both studies complement nicely each other. Together, they unequivocally confirm that aberrant DNA methylation at imprinted loci is a frequent event in human HCC, which might be exploited in the future as a new biomarker. The differences between both studies are mainly due to the fact that many genes are represented by only one or two CpG sites on the GoldenGate array, that not all regions analysed by us are represented on the Illumina array, and that our approach focused from the beginning not only on genes but also on imprint control regions.

Although moderate aberrant DNA methylation has been reported in some tumour suppressor genes in hepatocellular adenoma [40, 41], we do not observe any methylation aberration at imprinted loci in this benign liver tumour and also not in the benign proliferation focal nodular hyperplasia [27]. These results indicate that aberrant methylation at imprinted loci is a specific event in the malignant transformation of hepatocytes and not only a by-product of increased proliferation accompanied by reduced fidelity of DNA methylation maintenance.

Deregulation of DNA methylation at imprinted loci is obviously independent from generalized hypomethylation in repetitive sequences in HCC. As clearly discernible from Fig. 3, subtle loss of methylation at imprinted loci is accompanied by a substantial decrease of *LINE1* methylation levels whereas gain of methylation at these loci occurs in the context of unaltered *LINE1* methylation. Several genes required for protection and maintenance of imprinting have been identified, particularly *ZFP57* and *TRIM28/KAP1*. The protein products of these genes form a chromatin modifier complex and act specifically at the imprint control regions [42, 43]. Within this complex, ZFP57 recognizes the imprinting control regions and TRIM28 recruits DNMT1 to stabilize the imprinting marks [42, 44]. Ablation of ZFP57 and disruption of *TRIM28* lead to specific loss of methylation at the imprinting control regions in mice [45]. However, how loss of ZFP57 or *TRIM28* function is related to extensive hypomethylation in imprinted loci in cancer cells is currently unknown. Upregulation of TET family proteins that are involved in an active DNA demethylation process through the generation of 5-hydroxymethyl-cytosine has also been implicated in the development of human cancer [46]. *TET2* is also crucial for proper imprint regulation and might be involved in the observed widespread demethylation at imprinted loci in HCC [47]. Heterochromatin with H3K9me3 marks is able to recruit STELLA/PGC7 to maternal alleles and to some paternal DMRs protecting them from active demethylation by TET family proteins. Loss of PGC7 function is also likely to be an initiation event for generalized loss of DNA methylation at imprinted loci [48, 49].

Dysregulation of Wnt/β-catenin signalling has been implicated in liver carcinogenesis in which β-catenin mutations are observed in 20–30 % of HCCs [50–52]. *CTNNB1* mutations confer activation of the Wnt pathway leading to disturbance of cell-cell contacts and stimulation of cell proliferation and migration. The connection between *CTNNB1* mutations and frequent hypermethylation of tumour suppressor genes has been first described by Nishida et al. [25]. However, the molecular mechanism of this correlation remains unclear. In that study, 18 well-described tumour suppressor loci and three intergenic loci with unknown function were analysed using low-resolution semiquantitative COBRA methodology [53]. Imprinted loci were not studied by Nishida et al. In our HCC cohort, β-catenin is mutated more frequently in HCC specimens with loss of methylation at imprinted loci. This highlights important differences in the regulation of DNA methylation patterns at tumour suppressor genes and imprinted loci. Thus, the mechanism linking *CTNNB1* mutations and DNA methylation aberrations still needs further investigations.

## Conclusions

This study shows frequent and extensive DNA methylation aberrations at imprinted loci in human HCC but not in the adjacent liver tissues. In our HCC cohort, hypomethylation at imprinted loci correlates with global loss of DNA methylation (measured as *LINE1* methylation), frequent *CTNNB1* exon3 mutation, and shortened overall survival. Therefore, DNA methylation at imprinted control regions represents a promising new biomarker for detection, sub-classification, as well as prognosis in HCC.

## Methods

### Patient samples and cell lines

A collection of primary human liver specimens were obtained from 40 HCC, 10 HCA, and 5 FNH patients who underwent surgery at the Medizinische Hochschule Hannover, snap frozen in liquid nitrogen and subsequently stored at −80 °C. All patient samples were analysed anonymously following a protocol approved by the local ethics committee (“Ethik-Kommission der Medizinischen Hochschule Hannover”, head: Prof. Dr. Tröger). Verification of tumour cell content to be at least 70 % was accomplished by an experienced pathologist using serial reference sections from each snap frozen specimen. Basic clinicopathological variables of the patients are summarized in Additional file 6: Table S2. Seven HCC cell lines (HLE, HLF, HuH7, HepG2, Hep3B, SNU182, and SNU387) and two immortalized hepatocyte lines (THLE-2 and THLE-3) were obtained from American Tissue Culture Collection (ATCC, Rockville, MD, USA) and cultivated under conditions recommended by ATCC. The proper identity of all cell lines was validated using short tandem repeat (STR) profiling following the protocol provided by the German Collection of Microorganisms and Cell Cultures (DSMZ, Braunschweig, Germany). All experiments using cell lines were performed at sub-confluent cellular density allowing exponential growth.

### DNA and RNA extraction

Extraction of high molecular weight DNA from the snap frozen primary specimens was performed by digestion with proteinase K (Merck, Darmstadt, Germany) followed by phenol/chloroform purification (ROTI® Carl Roth GmbH, Karlsruhe, Germany) according to standard protocols. Total RNA was extracted using TRIZOL™ reagent (Invitrogen, Darmstadt, Germany) following the instruction by the manufacturer.

### Bisulfite conversion and methylation analysis

For DNA methylation analysis, a total of 1 μg genomic DNA was treated with sodium bisulfite using the EZ DNA Methylation Kit™ (Zymo Research, HiSS Diagnostics, Freiburg, Germany) according to the manufacturer’s protocol. For each PCR amplification, approximately 25 ng of the bisulfite modified DNA were used. DNA methylation analysis was performed employing pyrosequencing as described previously [54]. All primer sequences are listed in Additional file 7: Table S3. The DNA methylation level for a given gene in a sample was calculated as the mean of the individual methylation levels of all CpG dinucleotides under study obtained from two independent pyrosequencing runs. The software Pyro-Q-CpG™ (Qiagen, Hilden, Germany) was used for calculating DNA methylation levels of each individual CpG dinucleotide. “Hypermethylated” and “hypomethylated” are defined as methylation value above and below mean of the adjacent liver tissue plus two times the standard deviation, respectively (normal range = mean_adj_ ± 2 × StD) [40].

### *CTNNB1* mutation analysis

The presence of mutations in exon 3 of the *CTNNB1* gene was analysed in primary HCC specimens using primers as described before [55]. For the sequencing reaction, the GenomeLab™ DTCS Quick Start kit (Beckman Coulter, Krefeld, Germany) and GenomeLab™ Genetic Analysis System (Beckman Coulter, Brea, CA) were used following the manufacturer’s instructions without deviation.

### Statistical analysis

Cluster analysis of DNA methylation values at imprinted loci was performed using Qlucore Omics Explorer v2.2 (Qlucore, Lund, Sweden). For further statistical analyses, GraphPad Prism (version 5.01 for Windows, La Jolla, CA, USA) was used. The Mann Whitney *U* test was utilized to compare continuous variables and *χ*^2^ test for relationships between categorical variables. More than two groups were compared by one-way ANOVA adjusted to non-parametric conditions (Kruskal-Wallis test). Differences in variances between two groups were assessed by using the F-test. To compare survival of HCC patients, Kaplan-Meier curves were constructed and log-rank (Mantel-Cox) test was used. For those comparisons, *p* < 0.05 was considered as statistically significant.

## Additional files

Additional file 1: Table S1. Compilation of all *p* values for all statistical calculations performed for the data displayed in Fig. 1. Additional file 2: Figure S1. Global DNA methylation levels (measured by LINE1 methylation). Additional file 3: Figure S2. Cluster analysis of methylation patterns at imprinted loci in HCC using Illumina 27K methylation array from (A) Heidelberg *n* = 63) [23] and (B) Taiwanese cohort (*n* = 62) [24] shows subgrouping of HCC into three groups: hypermethylation and moderate and severe hypomethylation. Additional file 4: Figure S3. Global DNA methylation level in correlation to DNA methylation at imprinted loci. Additional file 5: Figure S4. DNA methylation analysis at imprinted loci in HCA, FNH, the adjacent liver tissues, and healthy liver samples. Additional file 6: Table S2. Clinopathological variables of all HCC, HCA, and FNH patients involved in this study. Additional file 7: Table S3. List of primers used in this study.
