# Supplementary material for: Loss of DNA methylation at imprinted loci is a frequent event in hepatocellular carcinoma and identifies patients with shortened survival
Source: Clin Epigenetics. 2015 Oct 15;7:110. doi: 10.1186/s13148-015-0145-6 (PMC4606497; doi:10.1186/s13148-015-0145-6)
Supplement: Additional file 2: Figure S1. — Global DNA methylation levels (measured by LINE1 methylation). [file 13148_2015_145_MOESM2_ESM.doc]

**
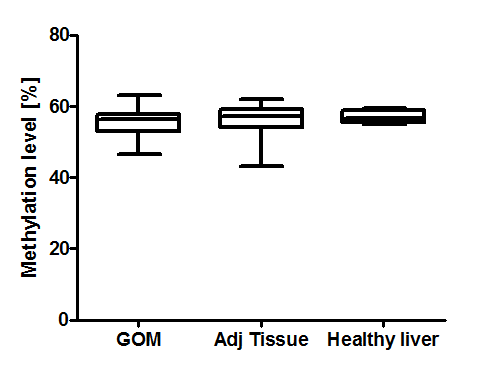
**

**Supplementary Figure S1.** *LINE-1* DNA methylation in HCC subgroup with imprint retention (hypermethylation) with the adjacent liver tissues and healthy liver tissues.
