# Supplementary figures and images for: Loss of DNA methylation at imprinted loci is a frequent event in hepatocellular carcinoma and identifies patients with shortened survival
Source: Clin Epigenetics. 2015 Oct 15;7:110. doi: 10.1186/s13148-015-0145-6 (PMC4606497; doi:10.1186/s13148-015-0145-6)

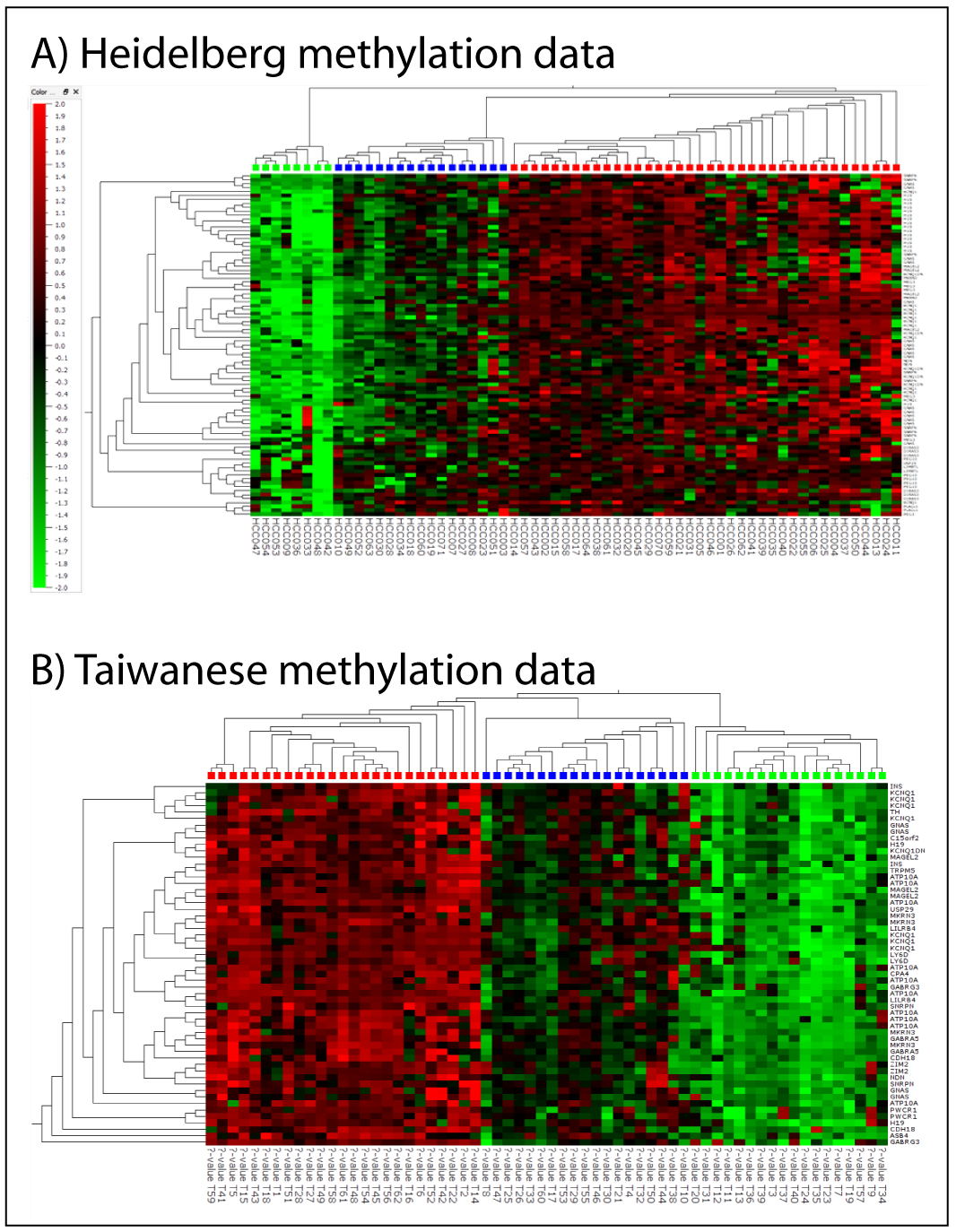

Supplement: Additional file 3: Figure S2. — Cluster analysis of methylation patterns at imprinted loci in HCC using Illumina 27K methylation array from (A) Heidelberg n = 63) [23] and (B) Taiwanese cohort (n = 62) [24] shows subgrouping of HCC into three groups: hypermethylation and moderate and severe hypomethylation. [file 13148_2015_145_MOESM3_ESM.tif]
