# Supplementary material for: Loss of DNA methylation at imprinted loci is a frequent event in hepatocellular carcinoma and identifies patients with shortened survival
Source: Clin Epigenetics. 2015 Oct 15;7:110. doi: 10.1186/s13148-015-0145-6 (PMC4606497; doi:10.1186/s13148-015-0145-6)
Supplement: Additional file 4: Figure S3. — Global DNA methylation level in correlation to DNA methylation at imprinted loci. [file 13148_2015_145_MOESM4_ESM.doc]

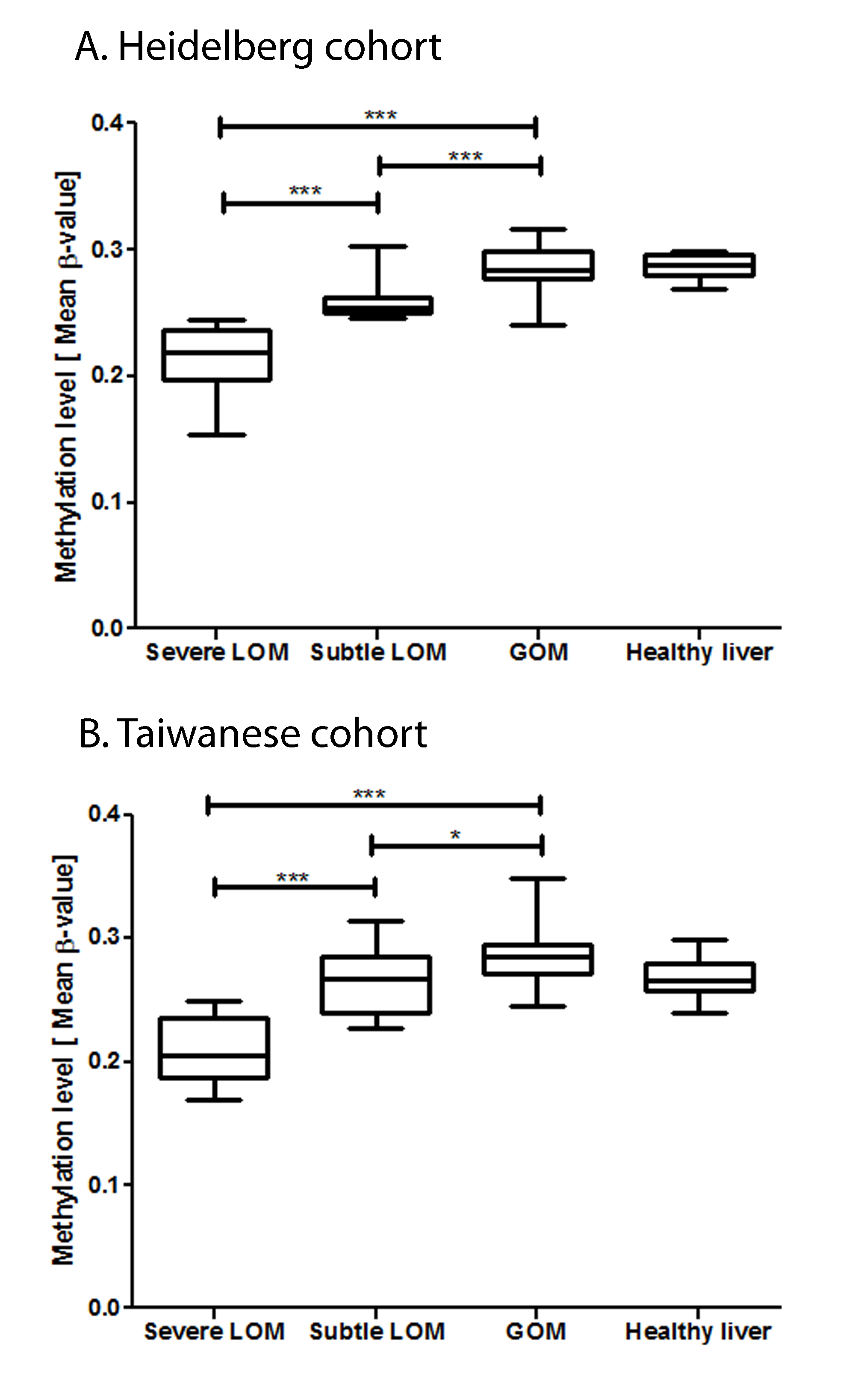


**Supplementary Figure S3.** Wide-spread loss of methylation in HCC subgroup with hypomethylation at imprinted loci.
