# Supplementary material for: Loss of DNA methylation at imprinted loci is a frequent event in hepatocellular carcinoma and identifies patients with shortened survival
Source: Clin Epigenetics. 2015 Oct 15;7:110. doi: 10.1186/s13148-015-0145-6 (PMC4606497; doi:10.1186/s13148-015-0145-6)
Supplement: Additional file 6: Table S2. — Clinopathological variables of all HCC, HCA, and FNH patients involved in this study. [file 13148_2015_145_MOESM6_ESM.docx]

**Supplementary Table S2.** Clinopathological variables of HCC, HCA, and FNH patients involved in this study

All primary specimens used in this study

HCC

| **HCC n=40** |  | **n** |
| --- | --- | --- |
| **Age** |  |  |
|  | <50 | 12 |
|  | >50 | 28 |
| **Sex** |  |  |
|  | Male | 33 |
|  | Female | 7 |
| **Etiology** |  |  |
|  | HBV | 8 |
|  | HCV | 4 |
|  | No infection | 28 |
| **Tumor differentiation** | |  |
|  | Good | 15 |
|  | Moderate | 17 |
|  | Poor | 8 |
| **Tumor size** | |  |
|  | <5cm | 20 |
|  | >5cm | 20 |
| **Stage** |  |  |
|  | I | 5 |
|  | II | 11 |
|  | III | 16 |
|  | IV | 8 |
| **Number of nodules** | |  |
|  | Unilocular | 14 |
|  | Multilocular | 26 |
| **Cirrhosis** |  |  |
|  | With Cirrhosis | 32 |
|  | Without Cirrhosis | 8 |
| **Survival** |  |  |
|  | <3 years | 18 |
|  | > 3years | 17 |
|  | No information | 2 |
|  | diagnosed <2y ago | 3 |

HCA and FNH

|  |  | **HCA (n=10)** | **FNH n=5** |
| --- | --- | --- | --- |
| **Age** |  |  |  |
|  | <50 | 10 | 4 |
|  | >50 | 0 | 1 |
| **Sex** |  |  |  |
|  | Male | 1 | 2 |
|  | Female | 9 | 3 |
| **Tumor size** | |  |  |
|  | <5cm | 4 | 4 |
|  | >5cm | 6 | 1 |
| **Number of nodules** | |  |  |
|  | Unilocular | 9 | 4 |
|  | Multilocular | 1 | 1 |
