# Supplementary material for: Loss of DNA methylation at imprinted loci is a frequent event in hepatocellular carcinoma and identifies patients with shortened survival
Source: Clin Epigenetics. 2015 Oct 15;7:110. doi: 10.1186/s13148-015-0145-6 (PMC4606497; doi:10.1186/s13148-015-0145-6)
Supplement: Additional file 7: Table S3. — List of primers used in this study. [file 13148_2015_145_MOESM7_ESM.docx]

**Supplementary Table S3.** List of primers used in this study

| **PRIMER** | **Forward** | **Reverse** | **Ta (⁰C)** | **MgCl2 (mM)** | **Sequencing** |
| --- | --- | --- | --- | --- | --- |
| **Pyrosequencing** | | | | | |
| **DIRAS3(3)** | AGTTTTGGTTTTAAGGAATAGAAGT | AACCCAACAACTAACAATAAATATTTTCA | 60 | 1.5 | AAGTTTTATAGGAAGATTAGAG |
| **DIRAS3(2)** | TGGATTAGTTTTTAGATTGTTGTAGATGT | CCCCAAAAACTCACTCCTCC | 60 | 1.5 | GGTTAGTTTTTTATAGTTGGT |
| **DLK1p** | TTYGGGATTTTAGYGATAAGTGTT | CRTTTAATACACRTTCCCTCACAC | 60 | 1.5 | GTTCCCTCACACTATACAAC |
| **GABRB3** | GYGGAGTGTGGGGAGAAG | ACCAAACTTCCRCRCTACC | 65 | 2.5 | GTGTGGGGAGAAGTAGTT |
| **GNAS-XL** | AGTGGGAGGAGGGGGTCCAGCCAAAG | ACTCCCCTGGCTAGGCTGGTGGGGTC | 60 | 1.5 | CCTCCAGGGAGAAAAGTG |
| **GRB10** | CTCTCCAAATACTCAAATAAACTC | GGTAGGGGTTTTTGTAGTTTG | 60 | 1.5 | CCAAATACTCAAATAAACTCC |
| **H19DMR** | YGGGGGTTTTTGTATAGTATATGG | ATATCCTATTCCCAAATAACCCC | 60 | 2.5 | GGTTTATYGTTTGGATG |
| **IGF2(0)** | AGGGGGTTTATTTTTTTAGGAAGTA | AACAAAAACCACTAAACACACAACTCTA | 60 | 2.5 | GGGTTTATTTTTTTAGGAAGTAT |
| **IGF2(2)** | GGTGGGTAGAGTAATTAGGGGA | CRCCTACRCAAAAACCTACCT | 60 | 1.5 | AGTAATTAGGGGAYGGTGA |
| **IG-DMR1** | ATTATTGAATTGGGTTTGTTAGTAG | CAAAACAACTCAAATCCTTTATAAC | 60 | 2.5 | TTAAAATATATCAAAAAACC |
| **IG-DMR2** | TAGYGATTTGTTAATTGYGAGTG | CRAATCCATTATAACCAATTACAATACCAC | 60 | 1.5 | CAATTACAATACCACAAAAT |
| **KCNQ1DN** | TGGAYGTTGGGYGTTTAAG | CCRCTACTCRCRACCTACA | 60 | 2.5 | GYGGAGGGTAGTAGGA |
| **KvDMR** | AGGGAAGTTTTAGGGTGTGAATTTTTAGAG | CCAAACCACCCACCTAACAAAAAAC | 60 | 1.5 | TGGTAATGTTTGGTATTT |
| **L3MBTL** | GAGTTGGTATGAGGYGAAGAGA | CCATCTTCRTATTCRATCACAC | 60 | 2.5 | TCRATCACACCTTCCTA |
| **MAGEL2** | GTGTTTATTTTTTTGATGGTTATAGTAG | CCAYGACTATCCTCTTAAACTTCC | 60 | 1.5 | TTTAAGAGGTTTTTAAGATT |
| **MEG3DMR1** | TTGGTTATYGGTYGTTTGAGG | AAAAAAATTCTACCRCAATACCCC | 60 | 2.5 | CAATACCCCTAACCRCCATAAC |
| **MEG3DMR2** | GTATTTTGATTTTTGYGAGAGGAT | ATCCCCACACACATACCCTTT | 60 | 2.5 | CTTTAAAAAAACCCCAA |
| **MEG3DMR3** | GTTYGTATTTTTYGATGGATGTT | CACCCTATAATCRCRAATACTTTT | 60 | 2.5 | ATAACTAACCRTCCTCAAAC |
| **MEST** | GTTTTGTGYGGGTTGTGGGTTG | CACCTACRCCCTAATCCCAAAAC | 60 | 1.5 | CGTTCGGTGTTTTGTAA |
| **MKRN3** | TAGTYGGGGGGGTTTTYGTTAT | ACCAAACCRAAACAACRACATT | 55 | 1.5 | AAAAAAAACTATCRCTATC |
| **NAP1L5** | AGTTYGGGTGTAGGGTTTTTG | TTCCRCCATTACCTCCTCT | 60 | 1.5 | YGGTTTTTTTTTTAATATGG |
| **NESP55** | TTAYGAGTAYGAGGAGGTAG | ACCRCAATCRTCTTCAAAC | 55 | 2.5 | TGTTTAGAGTAYGAGGAAGA |
| **NDN** | YGGGATYGTTTTTTTAGTAGGTGA | TACTACRCRCRACRCCTTC | 55 | 1.5 | TTTTTTTYGTYGGGG |
| **NNAT** | TGGYGGGYGGGTATTTAAG | CCRCCACTACCRCCATAATT | 60 | 2.5 | YGGTAGTGTGTTTAATAG |
| **NLRP2** | YGGTGTGTTGGATYGTGTGTA | TACACCCCRAACCTTTCCA | 60 | 1.5 | CRCRACCAAAAAACT |
| **PEG3** | GTGTAGAAGTTTGGGTAGTTG | AAACRCACTCACCTCACCTCAATA | 60 | 1.5 | ATTTTGGGTTGGTGG |
| **PEG10** | TTGGTTTAGGTGTGGGATTTT | AAACATTCTAAAATACTACTCCATCTC | 65 | 2.5 | TCCATCTCCCRCAACTCC |
| **RB1** | GGTAGGGTAGTTTTGGAAATGTTTAAG | AACCACAAACCCTTACCC | 60 | 1.5 | AGTTTTGGAAATGTTTAAGA |
| **SLC22A3** | YGYGTAAGGGTTAAGGGTTGG | RACCACTCCCRAAACTTCTAAAA | 60 | 1.5 | AAAACCCRATCTCTCA |
| **SNRPN** | GGAGTTGGGATTTTTGTATTG | ACCRCTCCTCAAACAAATAC | 60 | 2.5 | GTYGTAGAGGTAGGTTGG |
| **USP29** | AGGGTTATAAATGATTGTATTTGGG | AAAACAACTCRACCACAAACCCCC | 60 | 2.5 | TAAATGATTGTATTTGGGTT |
| **ZAC** | TGYGTTAGYGTTGTATTTGG | CRCRAAAAAAACRCTAAAACCCC | 65 | 2.5 | AACRCTAAAACCCCT |
| **ZBDF2** | TTTTGGGTAGTTTGGGGAGGA | CCCCCTTCAACCTAACAATACTCT | 60 | 2.5 | AAACAACAAACTCCACTAAT |
| **ZIM3** | AGGATTAGGTGGGGTTTTTGATA | AAACAAACAAAACCCAAACATTTA | 60 | 2.5 | TTTTGATTGGATTATGATG |
