# Supplementary material for: Loss of DNA methylation at imprinted loci is a frequent event in hepatocellular carcinoma and identifies patients with shortened survival
Source: Clin Epigenetics. 2015 Oct 15;7:110. doi: 10.1186/s13148-015-0145-6 (PMC4606497; doi:10.1186/s13148-015-0145-6)
Supplement: Additional file 5: Figure S4. — DNA methylation analysis at imprinted loci in HCA, FNH, the adjacent liver tissues, and healthy liver samples. [file 13148_2015_145_MOESM5_ESM.zip › Supplementary_FigureS4_Part2.doc]

**Supplementary Figure S4** (continued).
